# High-resolution 3D micro-CT imaging of breast microcalcifications: a preliminary analysis

**DOI:** 10.1186/1471-2407-14-9

**Published:** 2014-01-06

**Authors:** Inneke Willekens, Elke Van de Casteele, Nico Buls, Frederik Temmermans, Bart Jansen, Rudi Deklerck, Johan de Mey

**Affiliations:** 1 In vivo Cellular and Molecular Imaging Lab (ICMI), Vrije Universiteit Brussel (VUB), Brussels, Belgium; 2 Department of Radiology, Universitair Ziekenhuis Brussel, Brussels, Belgium; 3 Medische Beeldvorming en Fysische Wetenschappen (BEFY), Vrije Universiteit Brussel (VUB), Brussels, Belgium; 4 Vision Lab, iMinds, Universiteit Antwerpen, Antwerp, Belgium; 5 Department of Future Media and Imaging (FMI), iMinds, Ghent, Belgium; 6 Medical Image Computing, ESAT/PSI, iMinds, KU Leuven, Leuven, Belgium; 7 Future Health Department, iMinds, Ghent, Belgium; 8 Department of Electronics and Informatics (ETRO), Vrije Universiteit Brussel (VUB), Brussels, Belgium

**Keywords:** Breast cancer, Biopsies, X-ray micro-CT, Microcalcifications, 3D morphological analysis

## Abstract

**Background:**

Detection of microcalcifications on mammograms indicates the presence of breast lesion, and the shapes of the microcalcifications as seen by conventional mammography correlates with the probability of malignancy. This preliminary study evaluated the 3D shape of breast microcalcifications using micro-computed tomography (micro-CT) and compared the findings with those obtained using anatomopathological analysis.

**Methods:**

The study analyzed breast biopsy samples from 11 women with findings of suspicious microcalcifications on routine mammograms. The samples were imaged using a micro-CT (SkyScan 1076) at a resolution of 35 μm. Images were reconstructed using filtered back-projection and analyzed in 3D using surface rendering. The samples were subsequently analyzed by the pathology service. Reconstructed 3D images were compared with the corresponding histological slices.

**Results:**

Anatomopathological analysis showed that 5 of 11 patients had ductal breast carcinoma *in situ*. One patient was diagnosed with invasive ductal carcinoma.

Individual object analysis was performed on 597 microcalcifications. Malignant microcalcifications tended to be thinner and to have a smaller volume and surface area, while their surface area-to-volume ratio was greater than that of benign microcalcifications. The structure model index values were the same for malignant and benign microcalcifications.

**Conclusions:**

This is the first study to use micro-CT for quantitative 3D analysis of microcalcifications. This high-resolution imaging technique will be valuable for gaining a greater understanding of the morphologic characteristics of malignant and benign microcalcifications. The presence of many small microcalcifications can be an indication of malignancy. For the larger microcalcifications, 3D parameters confirmed the more irregular shape of malignant microcalcifications.

## Background

Breast cancer is the most common cancer among women worldwide [1] and ranks second in cancer-related deaths after colon cancer [2]. Early detection of suspicious lesions is thus crucial for the prognosis of the patient. Since Salomon’s radiographs of mastectomy specimens in 1913 [3], it has been known that microcalcifications are associated with breast cancer [4]. Gershon-Cohen *et al.*[5] were the first to report that the irregular, clustered appearance of calcifications was associated with breast cancer in 1962. Using radiology, Lanyi *et al.*[6] classified 5641 microcalcifications according to their shape, categorizing them as punctate, bean-shaped, linear, or branching. In 95% of cases, at least two of these configurations are identified simultaneously within individual clusters of microcalcifications (polymorphy).

State-of-the-art mammography is very sensitive in terms of detecting calcifications. However, calcifications can indicate benign or malignant lesions, leading to a large number of false-positive mammograms and a relatively low true-positive biopsy rate [7]. Since mammography is a projection radiography technique, it represents a 2D projection image of a 3D object. Consequently, superposition can result in underestimation of the number of microcalcifications. This can be overcome by 3D breast imaging techniques, such as breast computed tomography (CT) [8]. However, the resolution of breast CT is too low to resolve the 3D shape of microcalcifications.

The shape of microcalcifications is a major criterion for distinguishing malignant versus benign tissue. Thin linear, curvilinear, and branching shapes suggest malignancy, while round or oval shapes suggest benign lesions [9]. Clinical studies show that the shape of a microcalcification cluster and the spatial distribution of individual microcalcifications within it are important indicators of malignancy [10]. Note that in these studies, the microcalcification shape has typically been assessed using two 2D mammographic views.

The only way to determine the true shape of a 3D object is to use a 3D imaging technique. Some microcalcifications identified on mammograms have malignant characteristics, while others are non-specific. Thus, biopsy is required to establish a diagnosis. In an attempt to reduce the number of false-positives and unnecessary biopsies generated by screening mammography, radiologists have tried to define criteria that identify suspicious lesions and that help evaluate microcalcifications using properties such as microcalcification shape, size, clustering, location, and density [11]. Mammographic features that are commonly associated with malignancy include changes from a previous mammogram, distortion of the surrounding tissue architecture, association with less-dense tissue and calcifications, and the presence of more than ten calcifications in the lesion. The radiographic indications for breast biopsy include soft tissue lesion without calcifications, soft tissue lesion with calcifications, and a focus of calcifications without an associated soft tissue lesion [12]. Some investigators suggest that all patients with isolated microcalcification clusters at preoperative mammographic examination should have a localization biopsy due to the relatively high rate of malignant disease [13]. Because of the low radiographic contrast and the small size of the calcifications, methods have been sought that improve the detection of microcalcifications in mammographic images. A number of computer-assisted diagnostic (CAD) methods that use characteristics such as microcalcification size, shape, and distribution have been introduced to increase the accuracy of mammographic diagnosis [14-19].

Breast CT and tomosynthesis are newly developed breast imaging techniques, but they are still under investigation and currently are not commonly used in diagnostic imaging. These new techniques may outperform breast mammography for microcalcification detection [8], although they may not be better than mammography because of their spatial resolution. On the other hand, breast CT offers the advantage of three-dimensional anatomic detail, plus it eliminates superimposition of glandular tissues. However, in one study, screen-film mammography outperformed CT for visualization of microcalcifications [20] due to its higher spatial resolution. Other imaging techniques that have been explored for breast cancer detection included scintimammography [21], positron emission tomography [22], optical imaging [23], and microwave imaging [24]. Each approach has advantages, but so far, none have been able to compete with mammography as a screening modality [25].

The standard of care is routine mammography with additional ultrasound in glandular breasts or additional MRI. Taking a stereotactic core biopsy of indeterminate or suspicious breast calcifications using vacuum-assisted needles is a common practice. Radiography of these core samples is necessary to document the success of the procedure in extracting some of the target microcalcifications [26]. Afterwards, the biopsies are analyzed anatomopathologically. The pathologist tries to differentiate between benign and malignant tissue and, according to the cell type, determines the final diagnosis. Nishide *et al.*[27] reported that the resolution and contrast of micro-focus CT imaging is comparable to that of pathological images. Furthermore, microcalcifications were more clearly detected in micro-focus CT imaging than on the specimen radiographs.

In this study we used X-ray micro-computed tomography (micro-CT) to learn more about the actual 3D shape of microcalcifications in breast tissue. X-ray micro-CT is a non-invasive high-resolution imaging method that has been used primarily for bone imaging studies and material analysis. Micro-CT is also effective for contrast-enhanced soft tissue imaging [28,29]. This technique is increasingly used in a preclinical setting as an *in vitro* imaging method for analyzing tissue specimens and as an *in vivo* imaging method for evaluating small animals [30,31]. A literature search returned just one study [32] that used micro-CT to look at breast biopsies from 16 patients. However, that study focused on texture analysis rather than on shape. A second study used micro-CT to examine the structural and anatomic features of breast cancer specimens for intraoperative assessments [33,34].

The aims of this preliminary study were to evaluate the shape and number of breast microcalcifications using high-resolution X-ray micro-CT and to compare the findings with those obtained using radiography to analyze the breast specimens. The core samples containing microcalcifications were scanned, analyzed, and compared with the results of anatomopathological analysis. Although the sample size in this preliminary study was small (596 microcalcifications, 11 patients), it analyzes the relationship of the 3D shape of individual microcalcifications to malignancy based on high-resolution images.

## Methods

### Patient selection

In Belgium, women older than 45 years can have a routine mammography performed every 2 years. A radiologist who detects suspicious microcalcifications on a routine mammogram may decide to do a biopsy. The shape, size, clustering, location, and density of the microcalcifications are used to determine whether the findings are suspicious. Note that a microcalcification is defined as a calcification smaller than 1 mm; larger calcifications are termed macrocalcifications [35].

This study included the biopsy specimens of 11 women (age range, 46–78 years; mean age, 55 years) with suspicious microcalcifications on mammograms. The women were consecutive patients. The ethics committee at the university hospital (Commissie Medische Ethiek) approved the study. All patients provided informed written consent, which included consent for participation in the study and consent to publish the findings.

### Breast biopsy

Biopsies were performed by the Department of Radiology at the university hospital by an experienced mammographer. Minimally invasive vacuum-assisted stereotactic breast biopsies were performed using the Mammotome Biopsy System [Ethicon Endo-Surgery (EES), Inc., Johnson & Johnson, Langhorne PA, USA] under local anesthesia. The extracted samples had a diameter of 3 mm and a length of 23 mm and were put in a tube with formalin. Multiple samples (range, 8–21; mean number, 14) were taken from all 11 patients.

### X-ray Micro-CT Imaging

Each biopsy sample, in a tube with formalin, was scanned by micro-CT. Imaging was completed using a SkyScan 1076, an *in vivo* high-resolution X-ray micro-CT system with a rotating source-detector pair. This type of scanner has a fixed sample holder and was chosen to avoid movement of the biopsy samples, which were kept in liquid during scanning. The tubes containing tissue and formalin were placed on the isocenter of the scanner bed and stabilized using styrofoam.

The system consisted of a sealed 10-W micro-focus X-ray source with a 5-μm focal spot and a tungsten target that generates a broad polychromatic spectrum. To obtain optimal contrast in the breast biopsies, lower X-ray energies were selected by limiting the spectrum to 60 kV without hardware filtering, which would eliminate the soft X-rays from the spectrum. The large format X-ray detector (4000 x 2300) consisted of a gadolinium powder scintillator optically coupled with a tapered fiber to a cooled CCD chip. The projection images were taken every 0.5° and covered a 180° view with an exposure time of 1.8 sec. The total scanning time for each sample was 24 minutes. The projection images were resized in order to improve the signal-to-noise ratio, resulting in a voxel size of 35 μm. The reconstruction was performed using a modified Feldkamp cone-beam algorithm (NRecon, SkyScan/Bruker microCT, Kontich, Belgium) to return a stack of 2D cross-sectional images.

### Image analysis

The stack of micro-CT images was analyzed using the SkyScan analysis software package CTAn and 3D Calculator [36]. The analysis consisted of 3 steps: (1) segmentation of the microcalcifications from the background; (2) calculation of morphological parameters; and (3) generation of 3D models for visual inspection.

Segmentation was improved by using an automated local thresholding technique that was described by Waarsing *et al.*[36]. After segmentation, the following structural parameters were calculated for each segmented microcalcification of each patient: object volume (Obj.V), object surface (Obj.S), object surface/volume ratio (Obj.S/Obj.V), structure thickness (St.Th), structure model index (SMI), and the number of objects (Obj.N). The 3D volume was calculated based on the marching cubes model [37]. The Obj.S/Obj.V provides a measure of the smoothness of the particles. The St.Th, which was computed using a local sphere-fitting method [38], is a 3D measure of the average thickness of the microcalcifications. The SMI, a topological index, was calculated using differential analysis of the triangulated surface of the structure, which gives an estimate of the ratio of the number of plates to the number of rods that make up the 3D structure [39]). The SMI values range from 0 to 4, with 0 indicating an ideal plate-like structure, 3 an ideal rod-like structure, and 4 an ideal sphere. Intermediate values signify a mixed structure.

The structural parameters were first averaged over all microcalcifications per subject and compared between the benign and malignant group. Next, the individual microcalcifications associated with benign versus malignant lesions were analyzed. One object segmented in the samples from subject “Benign 2” was excluded from the analysis as its maximum diameter exceeded 1 mm and the object therefore did not qualify as a microcalcification [35].

Surface-rendered 3D models were made from the segmented datasets for visual inspection using CTAn/CTVol (SkyScan/Bruker microCT).

### Specimen radiography

The specimens were radiographed using a mammography system (GE Healthcare Senographe DS), 100-μm detector element at 25 kVp with a Mo anode-Mo filter combination. The beam quality was equal to 0.323-mm Al equivalent. These images were used to identify biopsy samples with and without microcalcifications.

For each radiograph, three expert mammographers, each with 5 to 15 years of experience, counted the number of microcalcifications in 2 reading sessions that were separated by an interval of 3 months to eliminate learning effects.

### Statistical analysis

The data were analyzed using IBM SPSS-Statistics version 20. Because of the low number of samples (5 benign, 6 malignant), non-parametric tests were used to compare the morphological parameters at the subject level between the two groups. The difference in the detection of microcalcifications by expert analysis versus micro-CT was assessed by the Wilcoxon signed-rank test (asymptomatic method). For the analysis of morphological parameters at the microcalcification level (414 microcalcifications associated with malignant lesions and 183 associated with benign lesions), the normality of the data was tested using the one-sample Kolmogorov-Smirnov test. For normally distributed data, the independent sample t-test was used to compare group averages; otherwise, the Mann–Whitney U test was used. The significance level in all tests was set to 0.05.

## Results

Anatomopathological analysis demonstrated that 5 of the 11 patients had ductal breast carcinoma *in situ* (DCIS). Of these, one patient had lower grade DCIS and 4 patients had high grade DCIS. Another patient was diagnosed with invasive ductal carcinoma.

The diagnoses of the patients with benign microcalcifications were fibrous breast tissue, benign fibroadenoma, fibrocystic disease, Reclus disease, and fibroadenoid hyperplasia. Table 1 summarizes the anatomopathological results for patients grouped according to benign or malignant diagnosis.

**Table 1:** Overview of the anatomopathological analysis and the amount of microcalcifications counted by experts and identified by X-ray micro-CT analysis

***Cases***	***Anatomopathological analysis***	***X-ray***		***X-ray***		***X-ray***		***Micro-CT***
		***Expert 1***		***Expert 2***		***Expert 3***		
		**R1**	**R2**	**R1**	**R2**	**R1**	**R2**	
Benign 1	Fibrous breast tissue with benign calcification	22	9	10	24	19	22	64
Benign 2	Calcified benign fibro adenoma + fybrocystic mastopathy	5	8	8	6	8	7	25
Benign 3	Fybrocystic disease	57	35	39	48	39	44	59
Benign 4	Disease of Reclus	18	10	10	10	16	14	15
Benign 5	Fibro adenoid hyperplasia	17	10	10	18	14	18	21
Malignant 1	DCIS grade 2 (cribriform type)	12	9	9	14	12	9	13
Malignant 2	High grade DCIS (cribriform type)	27	23	25	36	23	25	29
Malignant 3	High grade DCIS DIN3 (comedo type)	34	28	29	44	44	46	214
Malignant 4	High grade DCIS	47	31	30	51	49	52	110
Malignant 5	High grade DCIS with macrocalcifications (comedo type)	27	9	9	26	25	23	27
Malignant 6	Invasive ductal carcinoma	14	6	6	15	16	16	21

Ductal Carcinoma in Situ (DCIS); R1: reading 1; R2: reading 2.

**Figure 1:**
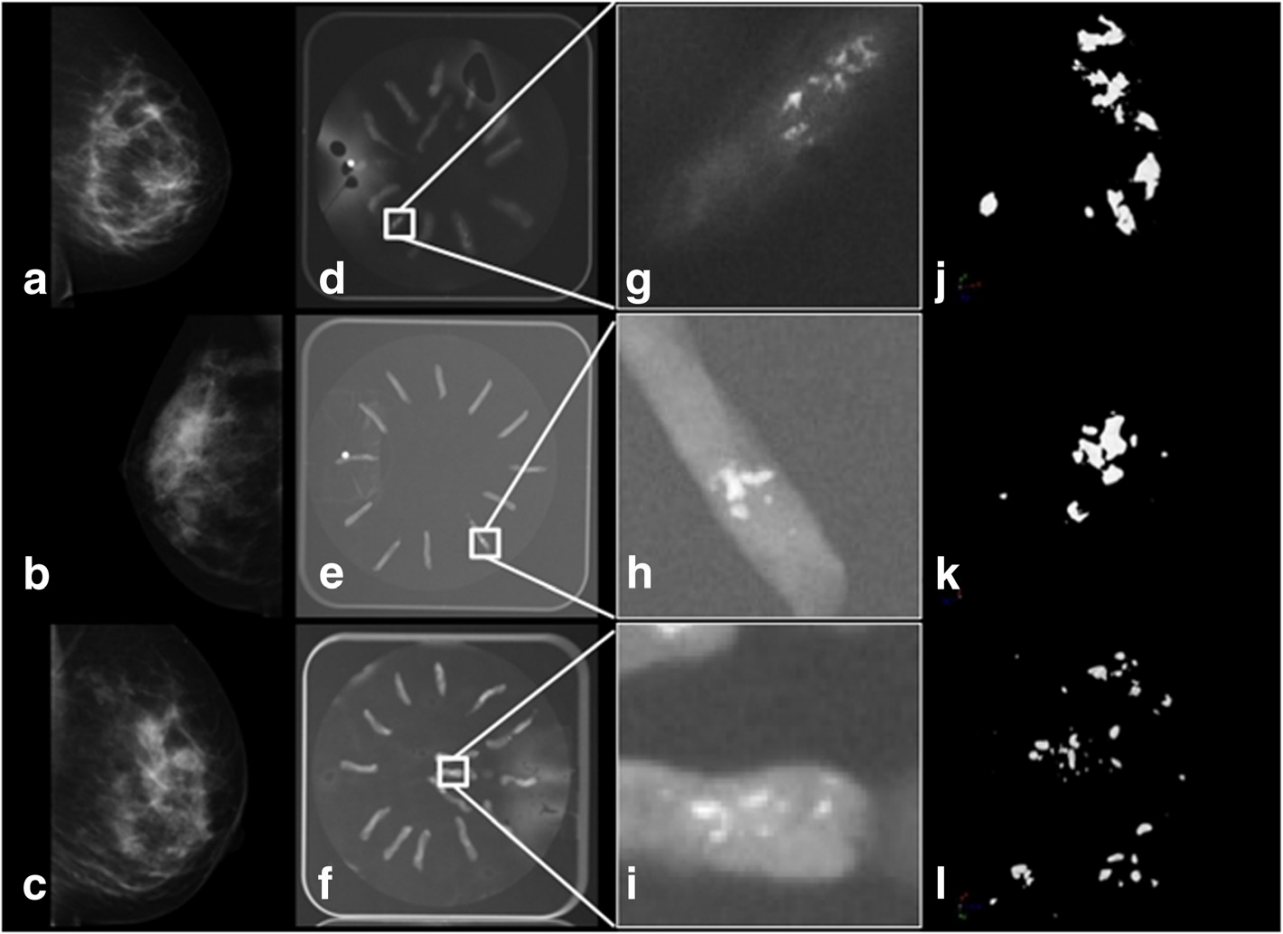
**Analysis of microcalcifications in benign tissue. (a-c)** Mammograms (cases: benign 1, 2, 3). **(d-f)** Radiographs of the breast core biopsies (placed on a dish). **(g-i)** Higher magnification of the specimen radiograph. **(j-l)** 3D micro-CT surface rendering of fibrous breast tissue (1), calcified fibroadenoma (2), and fibrocystic disease (3).

**Figure 2:**
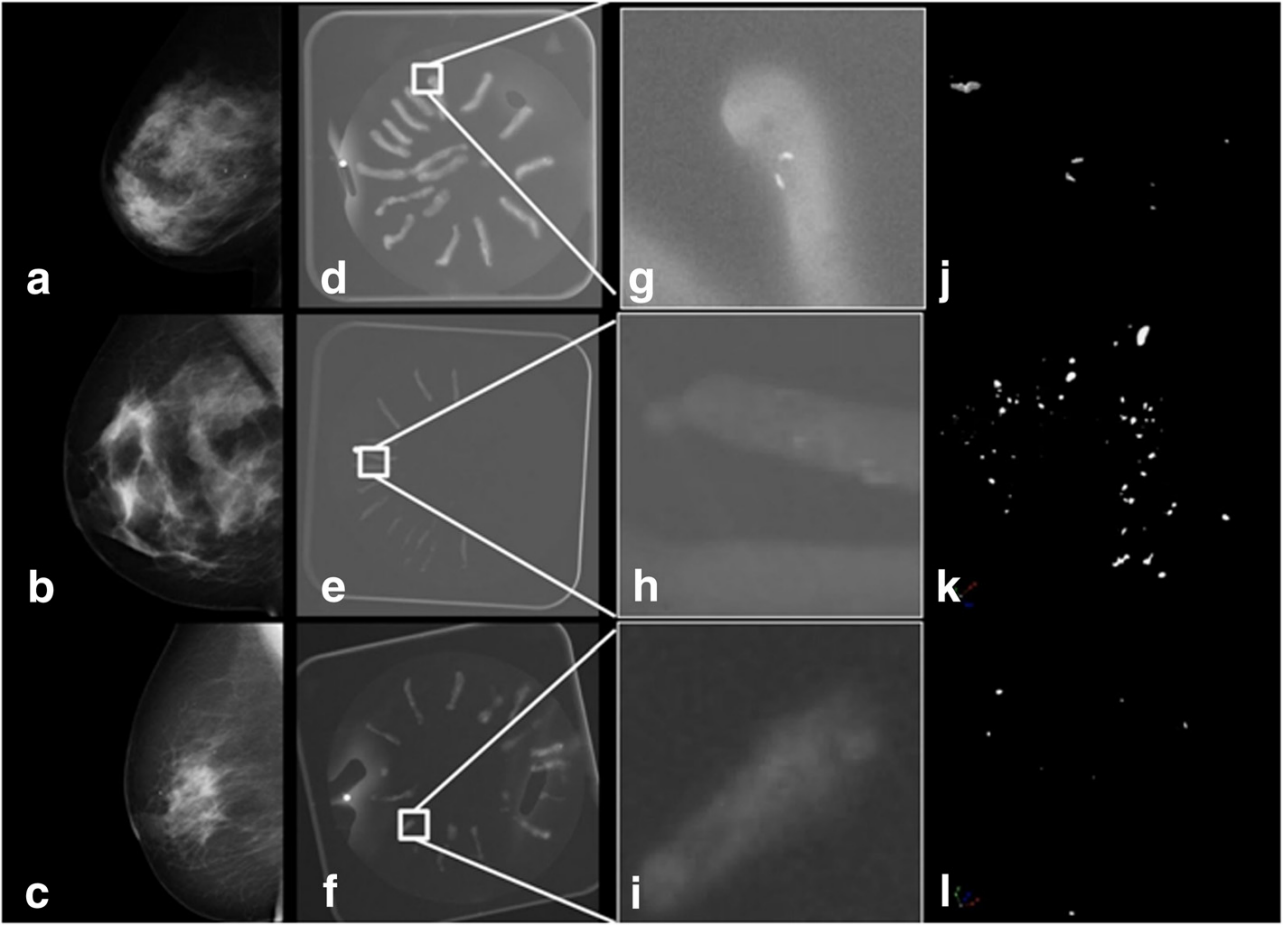
**Analysis of microcalcifications in malignant lesion. (a-c)** Mammograms (cases: malignant 1, 3, 6). **(d-f)** Radiographs of the breast core biopsies (placed on a dish). **(g-i)** Higher magnification of the specimen radiograph. **(j-l)** 3D micro-CT surface rendering of DCIS (1, 3) and invasive ductal carcinoma (6).

Suspicious microcalcifications are shown for 6 cases in Figure 1a–c (benign cases) and Figure 2a–c (malignant cases). Specimen radiography showed the presence of microcalcifications in the biopsy samples obtained by vacuum-assisted stereotactic breast biopsy (Figures 1 and 2d–f). A higher magnification of the radiography image is shown in the 3^rd^ column of Figure 1 and in Figure 2g–i. Surface renderings of the microcalcifications, shown in Figure 1 and Figure 2j–l, illustrate the 3D nature of the micro-CT imaging technique.

**Figure 3:**
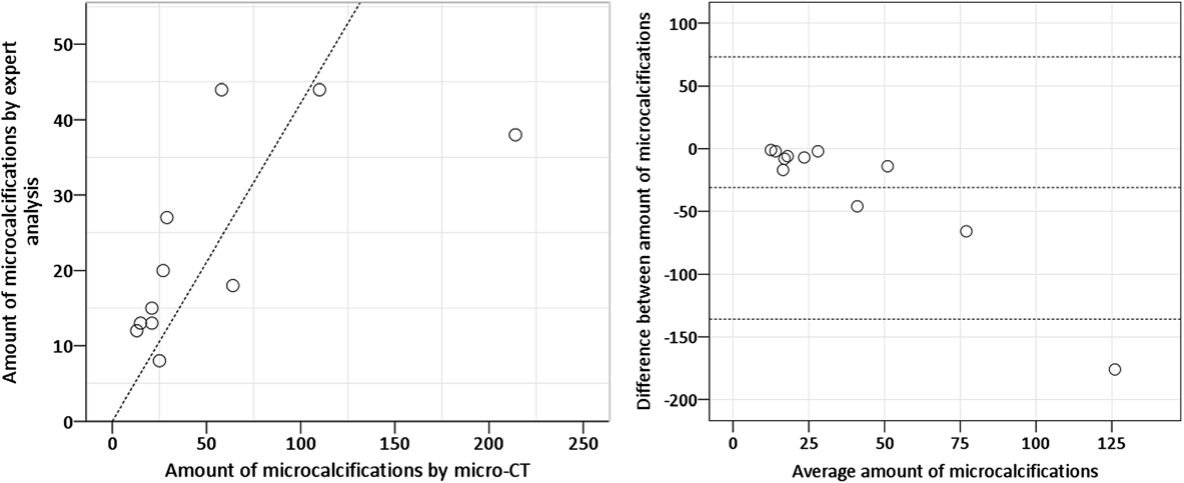
**Comparison of the number of microcalcifications detected by expert analysis versus micro-CT by regression analysis (left) and Bland-Altman plot (right).** The dotted lines on the Bland-Altman plot represent the average difference ± 2SD (standard deviation).

Table 1 shows the number of microcalcifications in the specimens as detected by expert analysis of the specimen radiographs shown in Figures 1 and 2d–f and the number detected by micro-CT analysis. Agreement between the two was determined by regression analysis and is shown by a Bland-Altman plot (Figure 3). Micro-CT analysis detected more microcalcifications in both benign (p = 0.043) and malignant cases (p = 0.028). The ratio between the number of microcalcifications detected by expert analysis and by micro-CT was 0.278 (95% Confidence Interval (CI): 0.15–0.41).

Table 2 summarizes the 3D morphological parameters as averaged for all microcalcifications in each patient. Compared to microcalcifications in patients diagnosed with malignant lesions, samples from patients diagnosed with benign lesions had a higher average volume (0.0159 ± 0.0071 mm^3^ versus 0.0078 ± 0.0095 mm^3^), a larger surface area (0.30 ± 0.10 mm^2^ versus 0.18 ± 0.16 mm^2^), a smaller surface area-to-volume ratio (77.68 ± 37.96 mm^-1^ versus 81.02 ± 19.96 mm^-1^), were thicker (0.26 ± 0.04 mm versus 0.19 ± 0.04 mm), and had smaller SMI values (3.02 ± 0.11 versus 3.05 ± 0.06). Patients diagnosed with benign lesion had fewer microcalcifications, 36.60 ± 22.03, than those with malignant lesion, 69.00 ± 79.39. However, apart from thickness, none of these differences were significant.

**Table 2:** Overview of the 3D morphological parameters analysed as averages over all microcalcifications per patient

***Cases***	***Obj.V (mm***^***3***^ ***)***	***Obj.S (mm***^***2***^ ***)***	***Obj.S/Obj.V (1/mm)***	***St.Th (mm)***	***SMI***	***Obj.N***
Benign 1	0.0150	0.28	142.86	0.27	2.88	64
Benign 2	0.0237	0.37	59.53	0.30	3.69	24
Benign 3	0.0203	0.37	51.01	0.29	3.13	59
Benign 4	0.0050	0.14	79.07	0.20	3.07	15
Benign 5	0.0156	0.34	55.93	0.22	2.96	21
Mean ± SD	0.0159 ± 0.0071	0.30 ± 0.10	77.68 ± 37.96	0.26 ± 0.04	3.02 ± 0.11	36.60 ± 23.03
Malignant 1	0.0269	0.49	84.85	0.26	2.94	13
Malignant 2	0.0075	0.20	46.46	0.20	3.10	29
Malignant 3	0.0024	0.08	99.66	0.18	3.05	214
Malignant 4	0.0041	0.13	77.26	0.18	3.10	110
Malignant 5	0.0028	0.10	76.80	0.15	3.08	27
Malignant 6	0.0031	0.10	101.12	0.17	3.03	21
Mean ± SD	0.0078 ± 0.0095	0.18 ± 0.16	81.02 ± 19.96	0.19 ± 0.04	3.05 ± 0.06	69.00 ± 79.39
Sign Man-U	0.126	0.126	0.662	0.017*	1.00	0.662

Object Volume (Obj.V) in mm^3^, Object Surface (Obj.S) in mm^2^, Object Surface/Object Volume (Obj.S/Obj.V) in 1/mm, Structure Thickness (St.Th) in mm, Structure Model Index (SMI), and Object Number (Obj.N). Bottom row gives the significance of group differences.

In addition to calculating the morphological parameters that describe the microcalcifications in each biopsy sample, individual object analysis was performed on microcalcifications that were larger than 2 image voxels (0.000086 mm^3^). Table 3 shows the mean and standard deviation of these individual results and of the number of objects found in benign and malignant samples classified according to their volume. The volume threshold was set at 0.0042 mm^3^, which corresponds to a microcalcification that might be visible on mammography, depending on the contrast. In the mammography images, the pixel size is 0.1 mm, so a microcalcification of 2 pixels corresponds to a volume of 0.0042 mm^3^ (slightly less than 100 voxels in the micro-CT images), assuming perfectly spherical objects with a diameter of 0.2 mm.

**Table 3:** Overview of the individual object analysis results divided in two groups representing lesions with a size on mammography of 0.2 mm or 2 mammography image pixels

		**Benign**	**Malignant**	**Test**	**Sign.**
					**P = 0.05**
	***Total Obj.N***	182	414		
**Objects ≤ 0.0042 mm**^**3 **^**(and larger than 2 micro-CT image voxels)**	***Obj.V (mm***^***3***^ ***)***	0.0011 ± 0.0010	0.0011 ± 0.0010	T-test	ns
	***Obj.S (mm***^***2***^ ***)***	0.0504 ± 0.0374	0.0515 ± 0.0360	T-test	ns
	***Obj.S/Obj.V (1/mm)***	80.00 ± 36.29	73.71 ± 29.43	Independent samples Mann-Whitney U test	ns
	***St.Th (mm)***	0.0879 ± 0.0277	0.0882 ± 0.0315	T-test	ns
	***SMI***	3.13 ± 0.23	3.16 ± 0.2	T-test	ns
	***Obj.N***	93 (51%)	262 (63%)		
**Objects > 0.0042 mm**^**3**^	***Obj.V (mm***^***3***^ ***)***	0.0474 ± 0.0530	0.0152 ± 0.0230	Independent samples Mann-Whitney U test	s
	***Obj.S (mm***^***2***^ ***)***	0.8218 ± 0.7996	0.3618 ± 0.3659	Independent samples Mann-Whitney U test	s
	***Obj.S/Obj.V (1/mm)***	22.86 ± 7.14	29.71 ± 5.71	T-test	s
	***St.Th (mm)***	0.2300 ± 0.0658	0.1684 ± 0.0375	T-test	s
	***SMI***	3.02 ± 0.27	3.08 ± 0.21	Independent samples Mann-Whitney U test	ns
	***Obj.N***	65 (36%)	96 (23%)		

Object Volume (Obj.V) in mm^3^, Object Surface (Obj.S) in mm^2^, Object Surface/Object Volume (Obj.S/Obj.V) in 1/mm, Structure Thickness (St.Th) in mm, Structure Model Index (SMI), and Object Number (Obj.N). The %-value = percentage of particles of the full amount of benign or malignant particles.

In volume this is represented by a sphere with diameter 0.2 mm: volume = 0.0042 mm^3^.

More microcalcifications (Obj.N) were detected in the biopsies of patients with breast carcinoma than in the biopsies of healthy patients, although this difference was not statistically significant (Table 2). After excluding microcalcifications smaller than 2 micro-CT voxels and grouping the remaining calcifications according to volume, as described above, 73.8% of the calcifications in the small-sized group were in samples from patients with malignant lesions, while 59.3% of those in the large-sized group were in samples from patients with malignant lesions. In terms of the distribution of all the small calcifications [(small-sized) + (microcalcifications < 2 micro-CT voxels)] in samples from malignant cases, 76.8% were small, while in samples from benign cases, 63.9% were small (Table 3).

Although the average total volume of the microcalcifications in the benign versus malignant groups was not significantly different (Table 2), the data in Table 3 show that there were more small calcifications in the malignant group than in the benign group. Furthermore, when the morphological parameters of the calcifications were analyzed as averages for all of the subjects in the two groups, significant differences were observed for microcalcifications with a volume larger than 0.0042 mm^3^: object volumes of 0.0474 ± 0.0530 mm^3^ in the benign group versus 0.0152 ± 0.0230 mm^3^ in the malignant group (p < 0.001); object surface area of 0.8218 ± 0.7996 mm^2^ versus 0.3618 ± 0.3659 mm^2^ (p < 0.001); object surface area-to-volume ratios of 22.86 ± 7.14 mm^-1^ versus 29.71 ± 5.71 mm^-1^ (p < 0.001); thickness of 0.2300 ± 0.0658 mm versus 0.1684 ± 0.0375 mm (p < 0.001). The SMI values, 3.02 ± 0.27 versus 3.08 ± 0.21 (p = 0.188), were not significantly different between the two groups (Table 3).

## Discussion

The aim of this study was to analyze the 3D characteristics of microcalcifications detected by mammography using micro-CT. Micro-CT is a high-resolution imaging modality that complements the use of mammography.

The calcifications seen on the mammograms in Figures 1 and 2 had suspicious characteristics, prompting biopsy. On the specimen radiographs, however, the benign calcifications seemed brighter and thus easier to detect, which could be because they are larger, there are more of them, or because they are denser compared with the calcifications found in malignant samples. However, the brightness was not quantified but was based on visual comparison of the images. The 3D micro-CT results showed that there was a significant difference in the size of the calcifications between the two groups of patients with benign or malignant findings. The results confirmed that the benign calcifications were, on average, bigger than the malignant calcifications, which could lead to better visibility on the specimen radiographs. The characteristics and number of microcalcifications is not always clear on clinical mammograms [40] and specimen radiographs [26]. Table 1 shows that the experts that counted calcifications on the specimen radiographs underestimated the number compared to the 3D object analysis using micro-CT. The underestimation was even greater on the specimen radiographs when the microcalcifications were organized in clusters (see Figure 1 to compare patients “Benign 1, 2” with “Benign 3” as the microcalcifications were more evenly distributed in the latter). Figure 3 confirms this by showing the agreement between the number of microcalcifications detected with micro-CT and the number counted by the experts. In the Bland-Altman plot, the smallest differences are in samples with a small number of microcalcifications. There is one outlier at the right bottom side of the plot, which can be explained by the large difference between the expert’s count and the count by micro-CT in the sample with the most microcalcifications (see Figure 2k “Malignant 3”). This confirms that it can be difficult to analyze microcalcifications on projection images. It also emphasizes that 3D imaging of microcalcifications provides an added value for studying the number and the shape and size of calcifications.

Histopathology is still the most sensitive method for diagnosing breast malignancy and has remained the gold standard for diagnosing breast carcinoma for a long time [41]. Invasive ductal carcinoma is the most common type of breast cancer, making up 70–80% of all breast cancer diagnoses. The most frequent type of breast cancer detected in screening is DCIS [3,42], which can be comedo, cribriform, or micropapillary types. In this study, 5 of the 11 patients were diagnosed with DCIS. Comedo carcinoma, which is the most aggressive type, was found in 2 patients, and 2 other patients were classified as having the cribriform type. The comedo type of DCIS is characterized by linear and branching (casting) calcifications, while calcifications in the cribriform (and micropapillary) types are more punctate and vary in size and shape. Among the various parameters used to assess the diagnostic significance of microcalcifications, irregular shape is the most indicative of carcinoma, with a predictive value of 80%; further, 88% of carcinomas with microcalcifications have irregularly-shaped calcifications [42]. This preliminary study had no patients with low grade DCIS. It would be interesting to compare such a group with benign cases to further evaluate the possible prognostic power of micro-CT.

**Figure 4:**
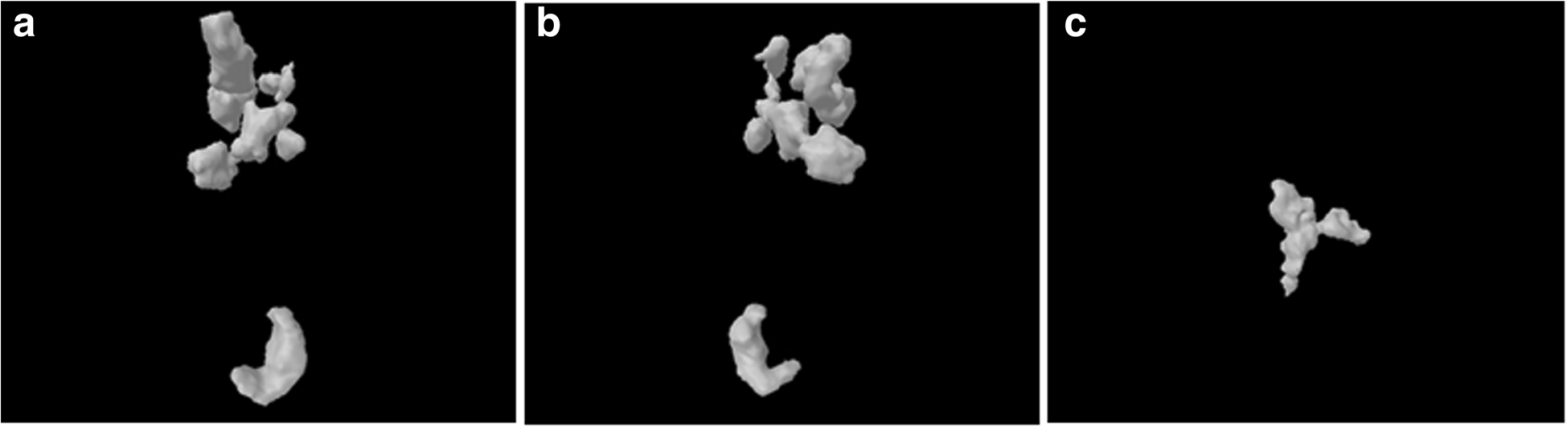
**3D models of microcalcifications. (a)** 3D model of the larger microcalcifications found in “benign 2”. The calcification at the bottom has a teacup shape as described in fibrocystic disease. At the top, a popcorn-like shape can be seen as described in fibroadenoma. **(b)** The same 3D model as in (a) viewed from another direction. **(c)** Example of a branching v-shaped microcalcification as a cribriform ductal carcinoma in-situ (“malignant 1”).

As described for the comedo and cribriform types of DCIS, different pathological entities can give rise to different calcification shapes. This also holds true for benign cases: Fibrocystic changes may give rise to ‘milk of calcium’ or teacup calcifications as seen in Figure 4a and b (bottom object), or to small calcifications organized in a cluster (Figure 1 (l)). Fibroadenoma may be associated with large popcorn-like calcifications (Figure 4a and b, top object).

In terms of malignant lesions, DCIS and invasive duct carcinoma can be associated with large irregular, rod or V-shaped, pleomorphic or branching-type calcifications that follow the distribution of the duct. This is seen clearly in Figure 4c. Furthermore, analysis of the characteristics of the calcifications can help predict tumor size, grade, and invasiveness [43]. The survival of women with masses or linear/linear-branching calcifications (i.e., casting calcifications) is considerably worse than the survival of women with other types of lesions, suggesting that the calcifications are associated with duct-forming invasive cancer [44]. Breast tumors associated with casting-type calcifications by mammography comprise a disease entity that exhibits substantially more aggressive behavior and poorer outcome than cancers with other mammographic features [45]. Microcalcifications associated with breast cancer are usually composed of hydroxyapatite, a bone-specific mineral. Histological examination of the lesions suggests that the microcalcifications are breast cancer cells that become mineralized. In other words, breast microcalcifications can be considered to be ‘fossils’ of cancer cells [46].

Radiologic-histologic correlations have shown that casting or rod-like calcifications in the duct are characteristic of malignancy. Malignant calcifications are typically more elongated, while benign calcifications are usually more round [47]. The microcalcification morphologic descriptors include coarse heterogeneous, amorphous, fine pleomorphic, and fine linear; these descriptors have a progressively increasing risk of malignancy [48]. Tables 2 and 3 show that the SMI values are around 3, indicating that the microcalcifications are cylindrical in both benign and malignant cases. The surface area-to-volume ratio is another important 3D parameter that describes the shape of an object. We found that this ratio was higher for the large particles in malignant cases (Table 3); these microcalcifications were pointier and rough, as reported in the literature.

In this study, the number of calcifications was not a relevant parameter for classification purposes, and it is only described as such in a few articles that seem to have contradictory results. Franceschi *et al.*[49] described a correlation between malignancy and the presence of more than 15 calcifications on mammography. In this study (Table 1), more than 15 calcifications/patient were seen in both groups, and the number of calcifications was not significantly different between the two groups (Table 2). Le Gal *et al.*[48] reported that the number of calcifications was higher in carcinomas, which we confirmed (Table 1, “Malignant 3 and 4”). For a given size, malignant microcalcifications tend to be more irregular, which corresponds to the most important clinical indications of malignancy i.e. linear or branching microcalcifications. Table 2, however, shows that the thickness of the benign calcifications in our study was significantly greater than in the malignant group. When we classified the individual microcalcifications according to their volume, 76.8% vs. 63.9% of the objects in the malignant and benign groups, respectively, were small (Table 3). Furthermore, there were 318 vs. 117 small calcifications in the malignant and benign groups, respectively; this included the smallest calcifications (< 2 image voxels). This relationship between malignancy and small-sized calcifications was also described by Franceschi *et al.*[49] and by Gufler *et al.* (30]. The latter study used micro-CT, although it did not focus on 3D volumes and shapes. The ability to view/detect these small calcifications is a clear advantage of using the high-resolution micro-CT system. The appearance of these smaller calcifications seems to be a feature of malignancy. The thickness and the object volume were also significantly different, with the benign group having larger structures than the malignant group. This might be why the physician decided to perform a biopsy in each of these patients, with the biopsy showing that these were false positives.

This preliminary study included only a small number of patients in order to lay the groundwork for a larger study. The samples are biased, since biopsies are only taken when malignancy is suspected. According to standard procedure, the specimen radiographs were not taken for diagnostic purposes but rather to classify the biopsies for embedding in paraffin. Therefore the radiograph settings were not correlated with the micro-CT settings.

Future studies should include more patients, ideally with different types of benign and malignant calcifications (including the 3 grades of DCIS) in order to statistically analyze the differences in the morphological parameters of these distinct groups. For larger microcalcifications, the 3D shapes should be characterized in greater detail. In terms of the smaller calcifications, additional parameters need to be investigated, such as the separation between particles as a descriptor for the cluster itself.

## Conclusion

Micro-CT shows promise as a valuable tool for understanding the morphologic characteristics of malignant and benign microcalcifications. Radiology and pathology, both of which are 2D techniques, can benefit from complementary 3D micro-CT evaluation of microcalcifications. In this preliminary study, micro-CT images of breast biopsies were related to the pathological diagnosis. The data showed that the appearance of many small microcalcifications can be an indication for malignancy. In larger microcalcifications, the 3D parameters confirmed the more irregular shape of malignant samples compared to benign samples.

## Abbreviations

3D: Three-dimensional; Micro-CT: Micro-computed tomography; 2D: Two-dimensional; CAD: Computed assisted diagnostic; CT: Computed tomography; MRI: Magnetic resonance imaging; EES: Ethicon endo-surgery; CCD: Charge-coupled device; CTAn: CT analyzer; Obj.V: Object volume; Obj.S: object surface; Obj.S/Obj.V: object surface/object volume; St. Th: Structure thickness; SMI: Structure model index; Obj.N: Object number; Mo: Molybdeen; DCIS: Ductal carcinoma in situ.

## Competing interests

The authors declare that they have no competing interests.

## Authors’ contribution

IW carried out the studies and drafted the manuscript. EVDC carried out the studies and drafted the manuscript. FT helped draft the manuscript. BJ helped draft the manuscript and performed statistical analysis. NB performed the statistical analysis. RD participated in the design of the study. JDM participated in the design of the study. All authors read and approved the final manuscript.

## Pre-publication history

The pre-publication history for this paper can be accessed here:

http://www.biomedcentral.com/1471-2407/14/9/prepub
